# Prevalence of Malnutrition Assessed by the GLIM Criteria and Association with Activities of Daily Living in Older Residents in an Integrated Facility for Medical and Long-Term Care

**DOI:** 10.3390/nu14173656

**Published:** 2022-09-04

**Authors:** Yoji Kokura, Ryo Momosaki

**Affiliations:** 1 Department of Nutritional Management, Keiju Hatogaoka Integrated Facility for Medical and Long-Term Care, Hosu 9270023, Japan; 2 Department of Rehabilitation Medicine, Mie University Graduate School of Medicine, Tsu 5148507, Japan

**Keywords:** nutritional status, nutrition disorders, residential facilities, nursing homes, insurance, long-term care, rehabilitation

## Abstract

Malnutrition is associated with poor functional outcomes in residents in long-term care facilities. The integrated facility for medical and long-term care (IFMLC) is a new Japanese long-term care facility where medical services can be provided. This study aimed to investigate the prevalence of malnutrition diagnosed based on the Global Leadership Initiative on Malnutrition (GLIM) criteria and its association with activities of daily living (ADL) in older residents in IFMLC. In this cross-sectional study of older residents, we diagnosed mild and severe malnutrition using the GLIM criteria and assessed ADLs using the Barthel index (BI). Multivariate regression analysis was used to investigate the relationship between BI score and GLIM-defined malnutrition. A total of 117 older residents (84 women; median age, 88 years) were analyzed in this study. The prevalence values of mild and severe malnutrition were 29% and 18%, respectively. Multivariate analyses for the BI score after adjusting for potential confounders showed that mild and severe malnutrition were independently associated with BI score (B = −6.113, *p* < 0.046; B = −8.411, *p* = 0.015, respectively). GLIM-defined malnutrition is negatively associated with ADLs in older residents in IFMLC.

## 1. Introduction

Malnutrition is a common problem often seen in older people receiving care in long-term care facilities (LTCFs). A systematic review found that 12–54% of the older people admitted to LTCFs were malnourished [1]. Malnutrition was consistently associated with cognitive impairment, depression, swallowing difficulty, and functional impairment [2]. A greater body mass index (BMI) was associated with a lower chance of mortality, whereas malnutrition primarily affects mortality in nursing home patients [2]. Therefore, the early detection and treatment of malnutrition should be addressed to maximize the functional capacity and quality of life of older adults in LTCFs.

Japan is the most aged country worldwide, and the burden of long-term care is continuously increasing. People aged ≥ 65 years accounted for 28.8% of the total population in 2020, and that proportion is expected to reach a peak of approximately 40% in 2040–2050 [3]. The Japanese public long-term care insurance system established an integrated facility for medical and long-term care (IFMLC) in 2018 to meet the needs of older people requiring nursing care, chronic care, and both medical and nursing care [4]. The IFMLC combines (i) routine medical management, (ii) end-of-life care and terminal care functions, and (iii) functions as a living facility [4]. In 2020, the IFMLC had 515 facilities with 32,634 beds, with many older people requiring nursing care during admission [5]. Meanwhile, 36.7% of Japanese IFMLC residents had a Barthel index (BI) score of 0 points [5]. Furthermore, the average BI score improvement during admission is negative for most of them [5], indicating that activities of daily living (ADL) decline is a problem at IFMLC.

In IFMLC, the association between the prevalence of malnutrition assessed using the Global Leadership Initiative on Malnutrition (GLIM) criteria and ADLs is unclear. The prevalence of malnutrition in LTCFs ranges from 1.5% to 66.5%, but definitions of malnutrition vary [2]. To tackle the issue of malnutrition in LTCFs, a consistent definition was needed. As a framework for diagnosing malnutrition in adults, the GLIM developed a set of evidence-based criteria in 2018 [6]. A diagnosis can be verified by any combination of one phenotypic criterion and one etiologic criterion under the GLIM criteria, which is based on the evaluation of three phenotypic and two etiologic criteria [6]. Phenotypic criteria include BMI, with criteria for each race and age group. Studies have reported the association between GLIM criteria severity classification and prognosis using BMI cutoff values for the Asian population [7,8]. However, no study has used Asian population cutoff values of BMI for older people in IFMLCs. To evaluate the accuracy of the diagnosis of malnutrition, several patient groups and illness situations will require appropriately powered, methodologically sound validation studies employing the full GLIM criteria.

This study aimed to investigate the prevalence of malnutrition diagnosed using the GLIM criteria and its association with ADLs in older residents in IFMLCs.

## 2. Materials and Methods

### 2.1. Study Design and Participants

This single-center study followed a retrospective cross-sectional design. The participating facility was the IFMLC Keiju Hatogawaka (Ishikawa, Japan), which provided rehabilitation services based on the Japanese insurance system. The study was conducted from 1 to 30 November 2021, and the study participants were people aged ≥ 65 years who were admitted to an IFMLC during the study period. All clinical data were obtained during the study period. Residents who died during the study period and data missing were excluded.

### 2.2. IFMLC

The basic policy of IFMLCs is to provide medical care management, nursing care, nursing care under medical supervision and other medical care, functional training, and daily living care to persons requiring long-term medical care based on the facility service plan [4]. In IFMLCs, various treatments, such as medications and tests, are performed based on a physician’s consultation. Terminal care is also performed. IFMLCs are equipped with examination rooms, treatment rooms, functional training rooms, clinical laboratories (where routine clinical tests can be performed on sputum, blood, urine, feces, etc.), X-ray imaging, and computed tomography [4]. Medical services include the management of enteral nutrition and total parenteral nutrition, sputum suctioning, oxygen therapy, tracheostomy care, ventilator management, insulin injections, and treatment of pressure ulcers [4]. The provision of medical services is a critical feature that distinguishes IFMLCs from other LTCFs.

The comprehensive team of an IFMLC is composed of physicians, care managers, care workers, nurses, pharmacists, physical therapists, occupational therapists, and registered dieticians. In IFMLCs, two full-time physical therapists and two full-time occupational therapists provide rehabilitation to residents. Rehabilitation consists of joint range of motion practice, basic movement practice, standing and walking training, ADL training, endurance practice, muscle strengthening practice, and a mental functioning approach. Within the first 3 months after admission, intensive rehabilitation is provided with the addition of short-term intensive rehabilitation.

In IFMLCs, one full-time registered dietitian provides nutrition care management. Nutrition care management is a Japanese long-term care insurance nutrition management method [9,10], and it includes nutrition assessment, nutrition care planning, and nutrition monitoring. On the basis of the nutrition care plan, the registered dietitian provides nutritional care, including the provision of appropriate food amounts and forms, nutritional counseling, and monitoring of nutritional intake and status to ensure that nutritional care is provided efficiently to each resident. Nutrition care is evaluated periodically for appropriateness, and changes are made to the nutrition care plan.

### 2.3. Data Collection

As part of routine evaluations, all clinical information was recorded. Relevant information was extracted from the medical records and retrospectively examined in this study. All clinical data were obtained during the study period. These data included the demographic characteristics of the participants (e.g., age and sex), affiliation before IFMLC admission, number of days spent at the IFMLC, primary diseases for facility admission, comorbidities, nursing care level (between 1 (mild) and 5 (severe) based on the assessment of care requirements) [11], weight loss before hospitalization, swallowing ability, nutritional status, muscle mass, rehabilitation dose (in minute/day) during the study period, and physical function. The effects of comorbidities were evaluated using the Charlson comorbidity index score [12], which was collected from electrical medical charts. The registered dietitian evaluated the swallowing ability using the Food Intake Level Scale (FILS) [13]. The FILS scores are based on the swallowing condition and can be scored subjectively, ranging from level 1 to level 10, with higher levels indicating greater swallowing ability. Levels 1–3, 4–6, and 7–10 indicate “no oral intake”, “oral intake and alternative nutrition”, and “oral intake alone”, respectively.

### 2.4. Malnutrition Assessment

The Malnutrition Universal Screening Tool (MUST) [14] was used for nutritional screening, and this was followed by an evaluation of malnutrition using the GLIM diagnostic criteria [6]. MUST has three ratings: acute sickness, weight loss, and BMI; the total scores range from 0 to 6 [14]. Low, medium, and high risk of malnutrition are indicated by scores of 0, 1, and 2, respectively. Hence, in this study, malnutrition was considered a risk with a MUST score of 1. The three steps of the GLIM criteria are as follows: first, patients at risk for malnutrition are identified using an established nutritional risk screening tool; second, malnutrition is identified in at-risk patients by identifying at least one phenotypic and one etiologic criterion; and third, the severity of malnutrition is assessed based on the phenotypic criterion.

First, in all patients, weight loss (≥5% weight loss in the previous 6 months and ≥10% weight loss in >6 months), a low BMI (<18.5 kg/m^2^ in patients aged < 70 years and <20.0 kg/m^2^ in patients aged ≥ 70 years), and reduced muscle mass (calf circumference (CC) < 34 cm in men and <33 cm in women) were employed to evaluate the phenotypic criteria. Weight loss was assessed using monthly weight measurements by nursing staff and caregivers using calibrated scales that allowed measurements while in a wheelchair. Data on weight loss in the past 6 months before admission were obtained by interviewing hospital staff, patients, or family members. BMI was calculated by dividing the body weight by the height squared (kg/m^2^). The cutoff value of the CC was taken from the sarcopenia diagnostic criteria for Asian populations [15], i.e., <34 cm for men and <33 cm for women, as assessed with dual-energy absorptiometry or corresponding standards evaluating other body composition measurements, such as bioelectrical impedance analysis. In this study context, CT and MRI were not accessible [6]. Registered dietitians measured anthropometric values on the nondominant or nonparalysed limb in the supine position. With the feet and ankles relaxed and the knee bent to 90 degrees, the biggest CC was measured using a regular measuring tape in 0.1-cm intervals.

Second, in all patients, reduced food intake (<50% of energy requirements for >1 week or any reduction for >2 weeks), assimilation problems (e.g., dysphagia, vomiting, and diarrhea), and disease burden/inflammatory condition (e.g., congestive heart failure, chronic obstructive pulmonary disease, chronic kidney disease, and cancer) were used to evaluate the etiologic criteria. Estimated energy requirements were established based on the activity level of each resident and the Dietary Reference Intakes for Japanese (2020) [16]. The energy adequacy ratio was calculated by dividing the mean energy intake by the estimated energy requirement. Food intake observed during the study period was obtained from residents’ medical charts. Using a visual estimating approach, nurses evaluated the quantity of food intake [17], and qualified dietitians further estimated the amount of nutritional intake.

Finally, malnutrition was classified as severe in individuals with at least one of the following: severe weight loss (>10% weight loss in the previous 6 months or >20% weight loss in >6 months), severely low BMI, and severely reduced muscle mass (CC < 24 cm in men and <21.4 cm in women). Patients were diagnosed with severe malnutrition (BMI < 17.0 and <17.8 for patients aged <70 and aged ≥70 years, respectively) based on the cutoff values reported previously for Asians [7]. Due to the lack of clear cutoffs for substantially reduced muscle mass, prior studies in Japan [18,19,20] were used to define severely reduced skeletal muscle mass as 4 standard deviations below the mean of healthy adolescents. Each step of the GLIM process, including the MUST, was performed by certified, trained, and registered dietitians.

### 2.5. Outcome Measurement

The outcome was measured by determining independence in performing ADLs using BI [21]. The total value of the ten items in BI ranges from 0 to 100, and include feeding, bathing, grooming, dressing, bowel incontinence, bladder incontinence, toilet use, transfers, mobility, and stairs. The degrees of functional ability for each BI category fall in a range, with the greatest values signifying complete independence and lower BI scores suggesting less physical function.

### 2.6. Sample Size Calculation

The standard deviation of the BI for older patients in nursing homes in Japan was 28.5 in a previous study [22]. At a statistical power of 0.8, it takes 64 participants to reject the null hypothesis that the population means of the two groups are identical, even if the real difference between the means of the malnutrition and nonmalnutrition groups is 10. With this null hypothesis test, the likelihood of a type I error is 0.05. Therefore, we planned to examine >128 participants in the study.

### 2.7. Statistical Analyses

Categorical variables are presented as numbers (percentages); parametric variables, as means ± standard deviation; and nonparametric variables, as medians (interquartile ranges (IQR), 25–75 percentile). The background characteristics for malnutrition using the GLIM criteria were compared using the chi-squared test, Fisher’s exact test, Student’s *t*-test, and Mann–Whitney U test. For BI over the study period, correlation analysis was performed using Spearman’s rank correlation coefficient. The factors that were independently related to BI were determined using a multivariate linear regression analysis. The covariates that were selected to adjust for bias were age, sex, primary diseases for facility admission, and the estimated time of rehabilitation dose [23,24,25,26,27]. Using the variance inflation factor (VIF) coefficient, multicollinearity was assessed. The multivariable analysis did not include a covariate if its VIF was less than 2. *p*-values of <0.05 were considered significant. JMP 11.2.1 software was used to conduct statistical analysis (SAS Japan, Tokyo, Japan).

## 3. Results

**Figure 1:**
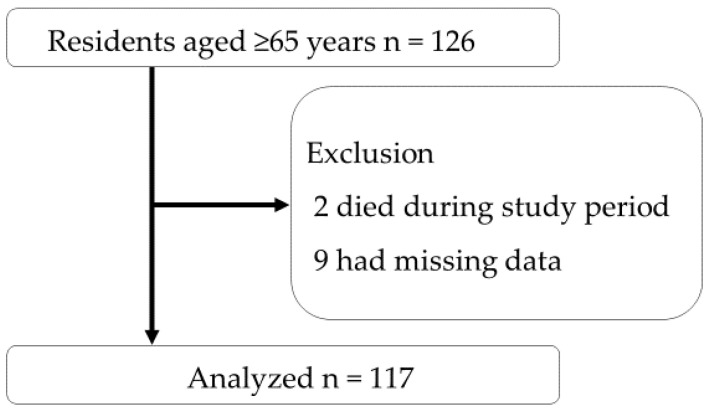
Flowchart of the study participants. The study analyzed 117 participants.

A total of 126 residents fulfilled the inclusion criteria. Of these, two died during the study period, and seven had missing data, which were excluded. Finally, there were 117 participants in total (33 men and 84 women, with an average age of 88 years) (Figure 1). The median number of days spent in IFMLCs was 483 (IQR: 207–1175) days.

The characteristics of the participants at the time of the survey are shown in Table 1. During the survey period, 34 (29%) residents were classified into the mild malnutrition group, and 83 were classified into the nonmalnutrition group. Moreover, 21 (18%) residents were classified into the severe malnutrition group, and 96 were classified into the nonmalnutrition group. A comparison of the proportion of affiliation before IFMLC admission between mild and severe malnutrition and nonmild and nonsevere malnutrition groups showed no significant difference (*p* = 0.709). In addition, a comparison of the proportion between the severe malnutrition and nonsevere malnutrition groups showed no significant difference (*p* = 0.445). A comparison of the proportion of primary diseases at IFMLC admission between the mild and severe malnutrition and nonmild and nonsevere malnutrition groups revealed a significant difference (*p* = 0.001). The most common affiliation before IFMLC admission for both the mild and severe malnutrition groups was a hospital. Moreover, a comparison of the proportion between the severe malnutrition and nonsevere malnutrition groups showed a significant difference (*p* = 0.022). The mild and severe malnutrition groups had a significantly higher proportion of dementia and heart failure than the nonmalnutrition group. The mild and severe malnutrition group had a significantly higher proportion of dysphagia than the nonmild and nonsevere malnutrition group (*p* = 0.016). The severe malnutrition group had a significantly lower BMI, higher proportion of the high risk of MUST, and more dysphagia than the nonsevere malnutrition group (all *p* < 0.05).

Table 2 indicates the nutritional characteristics based on the GLIM components in the malnutrition and nonmalnutrition groups and the BI. The prevalence of reduced food intake or assimilation disease burden/inflammation was significantly higher in the mild and severe malnutrition groups than in the nonmalnutrition group, respectively. No differences were found in the BI between the mild and severe malnutrition groups and the nonmalnutrition group.

The univariate analysis revealed no significant difference in the BI between the groups according to sex and primary diseases for facility admission (Table 3). The correlation analysis showed a significant correlation between BI and CCI (ρ = −0.195; *p* = 0.035) and estimated time of rehabilitation dose (ρ = 0.341; *p* < 0.001). Table 4 shows the results of the multivariate linear regression analyses for BI after adjusting for potential covariates.

The variables did not exhibit any multicollinearity. The multivariate analyses showed that GLIM-defined mild malnutrition (B = −6.113; 95% confidence interval (CI) = −12.129 to −0.098, *p* = 0.046) was independently associated with BI. GLIM-defined severe malnutrition (B = −8.411; 95% CI = −15.137 to −1.684, *p* = 0.015) was also independently associated with BI (Table 4).

## 4. Discussion

We conducted a cross-sectional study of geriatric IFMLC residents to determine the prevalence of malnutrition assessed by the GLIM criteria or its relationship with ADLs. Two important findings from the study were related to nutritional support for older IFMLC residents. First, the prevalence values of mild and severe malnutrition were 29% and 18%, respectively. Second, GLIM-defined mild and severe malnutrition were associated with ADL. To our knowledge, this study is the first to mention the prevalence of GLIM-defined malnutrition in older residents in IFMLC or its association with ADLs.

In this study, the prevalence values of mild and severe malnutrition were 29% and 18%, respectively. In previous studies, the prevalence values of older people with GLIM-defined malnutrition in hospitals, rehabilitation facilities, nursing homes, and communities assessed were 16.3–46% [28,29], 52–66.9% [30,31], 10.5–17% [32,33], and 12.6%–24.4%, respectively [34,35]. Thus, IFMLCs may have similar or greater malnutrition than nursing homes. The mild and severe malnutrition groups had a significantly higher proportion of dementia, heart failure, and EN than the nonmalnutrition group. Dementia reflects high rates of dysphagia, and dysphagia is associated with nutritional deficits [36]. In addition, the mild and severe malnutrition groups had a significantly higher proportion of reduced food intake or assimilation than the nonmalnutrition group. EN among older people in LTCFs does not increase nutrient absorption to improve nutritional status [37]. Therefore, dysphagia likely made oral intake difficult, resulting in reduced food intake and, thus, low nutritional status. In addition, heart failure can cause inflammation [38]. Because patients with chronic diseases from hospitals are admitted to IFMLCs, where healthcare services are available, malnutrition is highly likely to be caused by invasion and inflammation due to chronic diseases. Therefore, starvation and inflammation appear to be the causes of malnutrition in IFMLC residents.

GLIM-diagnosed mild and severe malnutrition were associated with ADLs. A study reported the relationship between malnutrition and decreased functionality in older adults undergoing rehabilitation [39]. Specifically, GLIM-defined malnutrition was found to be associated with decreased physical performance in patients with cardiovascular disease [40], dementia [41], and stroke [42]. The results of the present study support these previous findings. The severe malnutrition group had a significantly higher proportion of low BMI and reduced muscle mass than the nonmalnutrition group. Sarcopenia, including muscle mass reduction, is more likely to occur in nursing homes, where people spend more time in bed and are often unable to choose what they eat, compared with community residents who are physically active and can choose their food [23]. Low muscle mass was associated with worsening ADLs [43]. Furthermore, CC was found to be significantly correlated with physical function [44]. Therefore, the finding that GLIM-defined severe malnutrition, including loss of muscle mass, is associated with decreased ADLs is reasonable. IFMLCs require improvement of malnutrition, including loss of muscle mass.

Rehabilitation nutrition practices may be effective in improving malnutrition in IFMLCs. It is defined as the combination of rehabilitation and nutrition care [45]. In this concept, nutritional management and rehabilitation are combined using the International Classification Guidelines on Dysfunction and Health to allow the patients to perform body functions, participate in activities, and achieve a quality of life by improving nutritional status, sarcopenia, and frailty. Enhanced nutritional care was weakly recommended for patients receiving rehabilitation for hip fractures and cerebrovascular disease to improve ADL [46]. Additionally, dietary supplements, giving residents more control over meal choices, and staff training programs were effective approaches to lower malnutrition in nursing homes [2]. Therefore, rehabilitation nutrition practices are recommended for patients in IFMLCs with malnutrition and decreased ADLs.

This study has several limitations. First, a causal relationship between malnutrition and reduced ADLs was unclear owing to the study design. The causal relationship between malnutrition and decreased ADLs must therefore be clarified through additional longitudinal research. Second, the outcomes of this observational study may not be as broadly applicable because it was restricted to a single IFMLC. Third, the small number of participants weakened the statistical power of this study. Thus, to overcome this limitation, multicenter prospective studies with a higher sample size are required.

## 5. Conclusions

In this study, the prevalence values of mild and severe malnutrition were 29% and 18%, respectively, in older patients in IFMLCs. The severe malnutrition group had a significantly higher proportion of low BMI and reduced muscle mass than the nonmalnutrition group. Starvation and inflammation appeared to be the causes of malnutrition in IFMLC residents. Furthermore, GLIM-diagnosed malnutrition was independently associated with ADLs in this population. Therefore, we suggest assessing malnutrition with a disability early and managing all IFMLC residents properly. Rehabilitation nutrition practices are recommended for patients in IFMLCs with malnutrition and decreased ADLs.

## Figures and Tables

**Table 1:** Resident characteristics during the survey period.

Characteristics	All (117)	Mild and Severe Malnutrition (34)	Nonmild and Nonsevere Malnutrition (83)	*p*-Value	Severe Malnutrition (21)	Nonsevere Malnutrition (96)	*p*-Value
Age (year), median (IQR)	88 (84–92)	89 (86–93)	87 (81–92)	0.120 ^(a)^	88 (86–93)	88 (82–92)	0.495 ^(a)^
Sex (female), *n* (%)	84 (72)	23 (68)	61 (74)	0.651 ^(b)^	15 (71)	69 (72)	1.000 ^(b)^
Affiliation before IFMLC admission, *n* (%)				0.709 ^(b)^			0.445 ^(b)^
Hospital	86 (74)	24 (70)	62 (74)		14 (67)	72 (75)	
Nursing home	13 (11)	5 (15)	8 (10)		4 (19)	9 (9)	
Home	18 (15)	5 (15)	13 (16)		3 (14)	15 (16)	
Spent at IFMLC, (days), median (IQR)	483 (207–1175)	416 (166–1004)	558 (211–1255)	0.189 ^(a)^	611 (230–1146)	479 (205–1179)	0.966 ^(a)^
Primary diseases for IFMLC admission, *n* (%)				0.001 ^(b)^			0.022 ^(b)^
Cerebrovascular disease	55 (47)	9 (26)	46 (56)		8 (27)	47 (54)	
Dementia	32 (27)	12 (35)	20 (24)		11 (37)	21 (24)	
Orthopedic diseases	9 (8)	4 (12)	5 (6)		4 (13)	5 (6)	
Heart failure	9 (8)	7 (21)	2 (2)		5 (17)	4 (5)	
Other diseases	12 (10)	2 (6)	10 (12)		2 (6)	10 (11)	
Nursing care level, *n* (%)				0.483 ^(b)^			0.467 ^(b)^
1	10 (8)	1 (3)	9 (11)		0 (0)	10 (10)	
2	19 (16)	5 (15)	14 (17)		2 (10)	17 (18)	
3	18 (15)	6 (18)	12 (14)		4 (19)	14 (15)	
4	34 (30)	13 (38)	21 (25)		8 (38)	26 (27)	
5	36 (31)	9 (26)	27 (33)		7 (33)	29 (30)	
CCI, (points), median (IQR)	2 (1–3)	2 (2–3)	2 (1–3)	0.130 ^(a)^	2 (2–3)	2 (1–3)	0.411 ^(a)^
BMI, (kg/m^2^), median (IQR)	19 (17–21)	17 (16–21)	19 (17–21)	0.148 ^(a)^	17 (16–17)	19 (17–22)	<0.001 ^(a)^
MUST, *n* (%)				0.103 ^(b)^			<0.001 ^(b)^
Low risk	22 (19)	5 (15)	17 (20)		0 (0)	22 (23)	
Medium risk	22 (19)	3 (9)	19 (23)		0 (0)	22 (23)	
High risk	73 (62)	26 (76)	47 (57)		21 (100)	52 (54)	
Dysphagia severity, *n* (%)				0.016 ^(b)^			<0.001 ^(b)^
Complete EN ^1^	20 (17)	5 (14)	15 (18)		4 (19)	16 (17)	
Oral intake with EN ^2^	6 (5)	5 (15)	1 (1)		5 (24)	1 (1)	
Oral intake without EN ^3^	91 (78)	24 (71)	67 (81)		12 (57)	79 (82)	
Estimated time of rehabilitation dose (minute/day), median (IQR)	9 (9–11)	9 (9–11)	11 (9–11)	0.678 ^(a)^	11 (9–11)	9 (9–11)	0.997 ^(a)^

^(a)^ Mann–Whitney U test and ^(b)^ Fisher’s exact test. IQR, interquartile range; IFMLC, integrated facility for medical and long-term care; CCI, Charlson comorbidity index; BMI, body mass index; MUST, Malnutrition Universal Screening Tool; EN, enteral nutrition. ^1^ Identified by Food Intake Level Scale 1–3. ^2^ Identified by Food Intake Level Scale 4–6. ^3^ Identified by Food Intake Level Scale 7–10.

**Table 2:** Nutritional characteristics based on the GLIM components in residents with and without malnutrition and the Barthel index.

Characteristics	Mild and Severe Malnutrition (34)	Nonmild and Nonsevere Malnutrition (83)	*p*-Value	Severe Malnutrition (21)	Nonsevere Malnutrition (96)	*p*-Value
Phenotypic criteria, presence, *n* (%)						
Weight loss	9 (26)	21 (25)	1.000 ^(a)^	3 (14)	6 (6)	0.203 ^(a)^
Low BMI by GLIM	24 (71)	55 (66)	0.650 ^(b)^	19 (90)	31 (32)	<0.001 ^(a)^
Reduced muscle mass	34 (100)	80 (96)	0.555 ^(a)^	8 (38)	7 (7)	<0.001 ^(a)^
Etiologic criteria, presence, *n* (%)						
Reduced food intake or assimilation	5 (15)	0 (0)	0.002 ^(a)^	3 (14)	2 (2)	0.040 ^(a)^
Disease burden/inflammation	32 (94)	1 (1)	<0.001 ^(a)^	19 (90)	14 (15)	<0.001 ^(a)^
Barthel Index (points), median (IQR)	13 (0–51)	15 (0–55)	0.672 ^(c)^	10 (0–38)	15 (0–55)	0.159 ^(c)^

^(a)^ Fisher’s exact test, ^(b)^ chi-squared test, and ^(c)^ Mann–Whitney U test. BMI, body mass index; GLIM, Global Leadership Initiative on Malnutrition; IQR, interquartile range.

**Table 3:** Univariate analysis of the Barthel index.

Characteristics	Barthel Index, Median (IQR)	*p*-Value
Sex		
Male	20 (5–58)	0.122 ^(a)^
Female	10 (0–50)	
Primary diseases for facility admission		
Cerebrovascular disease	10 (0–25)	0.06 ^(b)^
Dementia	15 (5–55)	
Orthopedic diseases	50 (13–70)	
Heart failure	55 (10–75)	
Other diseases	10 (0–35)	

^(a)^ Mann–Whitney U test and ^(b)^ Kruskal–Wallis test. IQR, interquartile range.

**Table 4:** Multiple linear regression analysis of the Barthel index.

Factor	*p*-Value	Β	95% CI	*p*-Value	Β	95% CI
Age	0.301	0.339	−0.307 to 0.986	0.403	0.270	−0.367 to 0.907
Sex, female	0.064	−5.620	−11.577 to 0.337	0.070	−5.430	−11.309 to 0.447
Primary diseases for facility admission						
Cerebrovascular disease	Reference			Reference		
Dementia	0.555	−2.998	−13.045 to 7.049	0.797	−1.306	−11.363 to 8.751
Orthopedic diseases	0.060	14.592	−0.949 to 30.133	0.044	15.935	0.472 to 31.399
Heart failure	0.060	15.415	−0.666 to 31.496	0.166	10.773	−4.549 to 26.095
Other diseases	0.040	−14.262	−28.131 to −0.393	0.069	−12.552	−26.074 to 0.970
Estimated time of rehabilitation dose (minute/day)	0.338	0.952	−1.008 to 2.912	0.314	0.988	−0.948 to 2.923
Mild and severe malnutrition	0.046	−6.113	−12.129 to −0.098	—	—	—
Severe malnutrition	—	—	—	0.015	−8.411	−15.137 to −1.684

Model 1, R^2^ = 0.166, *p* = 0.010; Model 2, R^2^ = 0.182, *p* = 0.005. B, partial regression coefficient; CI, confidence interval; GLIM, Global Leadership Initiative on Malnutrition.

## Data Availability

Not applicable.
